# A Cysteine Zipper Stabilizes a Pre-Fusion F Glycoprotein Vaccine for Respiratory Syncytial Virus

**DOI:** 10.1371/journal.pone.0128779

**Published:** 2015-06-22

**Authors:** Guillaume B. E. Stewart-Jones, Paul V. Thomas, Man Chen, Aliaksandr Druz, M. Gordon Joyce, Wing-Pui Kong, Mallika Sastry, Cinque Soto, Yongping Yang, Baoshan Zhang, Lei Chen, Gwo-Yu Chuang, Ivelin S. Georgiev, Jason S. McLellan, Sanjay Srivatsan, Tongqing Zhou, Ulrich Baxa, John R. Mascola, Barney S. Graham, Peter D. Kwong

**Affiliations:** 1 Vaccine Research Center, National Institute of Allergy and Infectious Diseases, National Institutes of Health, Bethesda, Maryland, United States of America; 2 Department of Biochemistry, Geisel School of Medicine at Dartmouth, Hanover, New Hampshire, United States of America; 3 Electron Microscopy Laboratory, Cancer Research Technology Program, Leidos Biomedical Research, Inc., Frederick National Laboratory for Cancer Research, Frederick, Maryland, United States of America; University of Iowa, UNITED STATES

## Abstract

Recombinant subunit vaccines should contain minimal non-pathogen motifs to reduce potential off-target reactivity. We recently developed a vaccine antigen against respiratory syncytial virus (RSV), which comprised the fusion (F) glycoprotein stabilized in its pre-fusion trimeric conformation by “DS-Cav1” mutations and by an appended C-terminal trimerization motif or “foldon” from T4-bacteriophage fibritin. Here we investigate the creation of a cysteine zipper to allow for the removal of the phage foldon, while maintaining the immunogenicity of the parent DS-Cav1+foldon antigen. Constructs without foldon yielded RSV F monomers, and enzymatic removal of the phage foldon from pre-fusion F trimers resulted in their dissociation into monomers. Because the native C terminus of the pre-fusion RSV F ectodomain encompasses a viral trimeric coiled-coil, we explored whether introduction of cysteine residues capable of forming inter-protomer disulfides might allow for stable trimers. Structural modeling indicated the introduced cysteines to form disulfide “rings”, with each ring comprising a different set of inward facing residues of the coiled-coil. Three sets of rings could be placed within the native RSV F coiled-coil, and additional rings could be added by duplicating portions of the coiled-coil. High levels of neutralizing activity in mice, equivalent to that of the parent DS-Cav1+foldon antigen, were elicited by a 4-ring stabilized RSV F trimer with no foldon. Structure-based alteration of a viral coiled-coil to create a cysteine zipper thus allows a phage trimerization motif to be removed from a candidate vaccine antigen.

## Introduction

Respiratory syncytial virus (RSV) is a highly contagious member of the *Paramyxoviridae* family and responsible for substantial morbidity and mortality in infants and the elderly worldwide [1–3]. Currently no commercial vaccine is available [4–6]. Immunoprophylaxis with the monoclonal antibody palivizumab (Synagis), which binds to the RSV fusion (F) glycoprotein, can prevent serious disease [7, 8], illustrating that if potent F-directed antibodies could be elicited by vaccination, then broadly affordable protection against this pathogen might be realized.

The RSV F glycoprotein forms a heterotrimer of F_2_ and F_1_ subunits [9]. A type I fusion machine, the RSV F trimer undergoes substantial conformational rearrangement in transiting from pre-fusion to post-fusion conformations to mediate virus-cell membrane fusion [10]. The pre-fusion conformation is meta-stable [10–14], and virions observed by electron microscopy show a significant proportion of viral spikes in the post-fusion conformation [14]. Potent neutralizing RSV antibodies bind specifically to the pre-fusion F trimer (e.g. D25, 5C4, AM22, MPE8) [10, 15–17]. The structure of pre-fusion RSV F, stabilized in this conformation by the D25 antibody, enabled structure-based stabilization of the pre-fusion state [18]. The membrane-distal region was stabilized by disulfide (S155C-S290C; ‘DS’) and cavity-filling (S190F, V207L; ‘Cav1’) mutations, while the membrane-proximal region was held by a C-terminal T4 bacteriophage fibritin trimerization domain (foldon) that assisted in retaining the trimeric state of the antigen. The foldon comprises 30 amino acids that assemble into a stable β-propeller structure with a hydrophobic core [19, 20] and trimerizes rapidly following expression [21]. This foldon motif has been used to stabilize trimeric antigens from numerous pathogens including HIV-1, RSV and influenza [18, 22–29]. Together the DS-Cav1 mutations and T4 bacteriophage foldon facilitated the generation of a stable recombinant pre-fusion F protein trimer capable of eliciting high levels of RSV neutralizing antibodies in mice and macaques [18].

While DS-Cav1 with foldon was suitable for proof-of-principle, an ideal vaccine should contain only sequences of the target pathogen, and removal of the T4 bacteriophage fibritin may reduce off-target responses. Furthermore, the removal of non-RSV F motifs might promote a more focused humoral response to RSV F sites of neutralization. It has recently been demonstrated that antigens fused to the foldon trimerization domain or other trimerization domains can elicit foldon-specific antibody responses [30]. To engineer a pre-fusion RSV F trimer without the heterologous foldon, we designed interprotomer disulfides within a native RSV F coiled-coil. We retained the foldon domain to promote RSV F trimerization; however, we introduced a protease-cleavage site between the RSV F and foldon to allow for the enzymatic removal of this domain. Designs were developed with minimal residue variation from the native RSV F sequence, and surface inaccessible mutations within the core of the trimer were varied to attempt to preserve the antigenic characteristics of the pre-fusion RSV F trimer surface. A cysteine zipper, comprising four interprotomer disulfide-forming rings within an extended coiled-coil, stabilized the RSV F trimer in the absence of foldon. The cysteine-zipper stabilized RSV F trimer–with no foldon–elicited a level of RSV neutralizing activity similar to that of parent DS-Cav1.

## Materials and Methods

### Design of disulfide linked variants

The D25-bound pre-fusion RSV F (PDB ID 4JHW) molecule was used to design and model the initial disulfide-linking mutations between residues 512 and 513 with the leucine 512 carbon-β located at a distance of 6 Å from the leucine 513 carbon-β on the adjacent protomer. Molecular modelling of the extended α10 helix, at the C terminus of the RSV F ectodomain, was performed by alignment of the GCN4 coiled coil helices (PDB ID 1PIQ) [31] to the RSV F α10 helices (PDB ID 4MMV) [18], with adjustments for the change in pitch of the helices at the junction. Analysis of the coiled coil A-G positions was done by the program DrawCoil 1.0: http://www.grigoryanlab.org/drawcoil/ [32]. For the 4-ring BCDE and 5-ring ABCDE variants, due to the beginning of the transmembrane region starting at approximately residue 530, hydrophilic amino acids based on previous RSV F α10 heptad repeat sequence were used to extend the helix and to allow an additional cysteine ring to be introduced. The constructs contain a thrombin cleavage site (LVPR) between the α10 helix and the T4 foldon sequence flanked by a Gly-Gly flexible linker after RSV F and a Gly-Ser-Gly-Gly linker between the thrombin site and the foldon.

### Calculation of proportions of covalently linked trimers and closed-ring trimers

We enumerated possible combinations of disulfide bonds, for each of one to five possible disulfide rings, and computed the fraction of these combinations that corresponds to disulfides formed between each pair of helices and the fraction of these that corresponds to *cis*-circularization, *trans*-circularization and combinations thereof for each variant. By *cis*-circularization, we mean covalent ring formation between three helices achieved by three interhelical disulfide bonds formed by a ring system comprising of pairs of adjacent cysteine mutations. By *trans*-circularization, we mean covalent formation between three helices achieved by interhelical disulfide bonds, where the disulfide bonds are formed in separate ring systems.

### Protein expression, purification and removal of the foldon

Pre-fusion RSV F variants were expressed by transient transfection of Expi293F cells using TrueFect-Max (United BioSystems, MD). Culture supernatants were harvested 5 days post transfection and centrifuged at 10,000 g to remove cell debris. The supernatants were sterile-filtered, and the RSV F variants were purified by nickel and streptactin-affinity chromatography followed by size-exclusion chromatography. The foldon domain was removed from the variants by digestion with 1.6 U/ml restriction-grade thrombin (Novagen) overnight at room temperature. The RSV F glycoprotein with foldon removed was then purified by a second round of size-exclusion chromatography in PBS prior to analysis or immunization.

### RSV F antigenic characterization

A fortéBio Octet Red384 instrument was used to measure binding kinetics of RSV F foldon-removed variants to antibodies that target the pre-fusion form (D25, AM22, 5C4). All assays were performed with agitation set to 1,000 rpm in phosphate-buffered saline (PBS) supplemented with 1% bovine serum albumin (BSA) to minimize nonspecific interactions. The final volume for all solutions was 50 μl/well. Assays were performed at 30°C in tilted black 384-well plates (Geiger Bio-One). Motavizumab IgG (50 μg/ml) in PBS was used to load anti-human Fc probes for 300 s, which were then used to capture relevant RSV F variants. Typical capture levels for each loading step were between 1.4 and 1.5 nm, and variability within a row of eight tips did not exceed 0.1 nm for each of these steps. The nm unit is a measure of the change in the interference pattern of white light reflected from the surface of the biosensor tip compared to an internal reference. This was measured in real-time and correlated with a change in the thickness of bound molecules on the biosensor tip surface. This can also be defined as a change in response units measured in nm [33, 34]. Biosensor tips were equilibrated for 240 s in PBS + 1% BSA prior to loading RSV F variants. Biosensor tips were then equilibrated for 90 s in PBS + 1% BSA prior to measuring association with antigen binding fragments (Fabs) in solution (0.007 μM to 0.5 μM) for 300 s; Fabs were then allowed to dissociate for 300 s-1200 s depending on the observed dissociation rate. Parallel correction to subtract systematic baseline drift was carried out by subtracting the measurements recorded for a loaded sensor incubated in PBS + 1% BSA. Data analysis and curve fitting were carried out using Octet software, version 8.0. Experimental data were fitted with the binding equations describing a 1:1 interaction. Global and local analyses of the data sets assuming reversible binding (full dissociation) were carried out using nonlinear least-squares fitting allowing a single set of binding parameters to be obtained simultaneously for all of the concentrations used in each experiment.

### Physical stability of RSV F variants

To assess the physical stability of the pre-fusion conformation of designed RSV F glycoproteins under various stress conditions, we treated the proteins with a variety of pharmaceutically relevant stresses such as extreme pH, high temperature, low and high osmolarity, and repeated freeze/thaw cycles while at a concentration of 50 μg/ml. The physical stability of treated RSV F variants was evaluated by the preservation of antigenic site Ø after treatment as assessed by binding of the site Ø-specific antibody D25.

In pH treatments, the RSV F glycoprotein solution was adjusted to pH 3.5 and pH 10 with appropriate buffers and incubated at room temperature for 60 minutes and subsequently neutralized to pH 7.5. Temperature treatments were carried out by incubating the RSV F glycoprotein solutions at 50°C, 70°C and 90°C for 60 minutes in a PCR cycler with heated lid. In osmolality treatments, RSV F glycoprotein solutions originally containing 150 mM NaCl were either diluted with 2.5 mM Tris buffer (pH 7.5) to an osmolality of 10 mM NaCl or adjusted with 4.5 M MgCl_2_ to a final concentration of 3.0 M MgCl_2_. Protein solutions were incubated for 60 minutes at room temperature and then returned to 150 mM salt by adding 5 M NaCl or dilution with 2.5 mM Tris buffer, respectively, and concentrated to 50 μg/ml. The freeze/thaw treatment was carried out by repeatedly freezing RSV F glycoprotein solutions in liquid nitrogen and thawing at 37°C ten times. All RSV F glycoproteins were diluted to 40 μg/ml with PBS + 1% BSA, and their ability to bind D25 Fab was measured with an Octet instrument using the protocol described above. The degree of physical stability is reported as the ratio of steady state D25-binding level before and after stress treatment.

### Negative stain electron microscopy

Samples were adsorbed to freshly glow-discharged carbon-film grids, rinsed twice with buffer and stained with freshly made 0.75% uranyl formate. Images were recorded on an FEI T20 microscope with a 2k x 2k Eagle CCD camera at a pixel size of 1.5 Å. Image analysis and 2D averaging was performed with Bsoft [35] and EMAN [36].

### Mouse immunizations

All animal experiments were reviewed and approved by the Animal Care and Use Committee of the Vaccine Research Center, NIAID, NIH, under animal protocol 13–454, and all animals were housed and cared for in accordance with local, state, federal, and institute policies in an American Association for Accreditation of Laboratory Animal Care (AAALAC)-accredited facility at the NIH. Hybrid mice that were the first filial offspring of a cross between BALB/cJ females (C) and C57BL/6J males (B6) (The Jackson Laboratory) known as CB6F1/J at ages 6 weeks to 12 weeks were intramuscularly injected with RSV F immunogens at week 0 and week 3. The frozen RSV F variant immunogen proteins were thawed on ice and mixed with 5-fold w/w poly I:C (Invivogen) adjuvant (i.e. 10 μg RSV F, 50 μg Poly I:C per animal per immunization), with injections taking place within 1 h of immunogen:adjuvant preparation. No adverse effect from immunization was observed. Blood was collected at least three days before immunization, and at week two, week five and week seven post initial immunization.

### Viruses and cells

Viral stocks were prepared and maintained as previously described [37]. Recombinant mKate-RSV expressing prototypic subtype A (strain A2) F genes and the Katushka fluorescent protein were constructed as reported by Hotard *et al*.,[38]. HEp-2 cells were maintained in Eagle's minimal essential medium containing 10% fetal bovine serum (10% EMEM), supplemented with glutamine, penicillin and streptomycin.

### RSV neutralization assays

Sera were distributed as four-fold dilutions from 1:10 to 1:40,960, mixed with an equal volume of recombinant mKate-Respiratory syncytial virus expressing prototypic F genes from subtype A (strain A2) and the Katushka fluorescent protein, and incubated at 37°C for 1 h. Next, 50 μl of each serum dilution/virus mixture was added to HEp-2 cells, which had been seeded at a density of 1.5x10^4^ in 30 μl MEM (minimal essential medium) in each well of 384-well black optical bottom plates, and incubated for 20–22 h before spectrophotometric analysis at 588 nm excitation and 635 nm emission (SpectraMax Paradigm, Molecular Devices, CA). The IC_50_ for each sample was calculated by curve fitting and non-linear regression using GraphPad Prism (GraphPad Software Inc., CA).

Protective threshold was calculated as follows: clinical administration of Palivizumab (Synagis) at 15 mg/kg, leads to patient sera levels at trough of ~40 μg/ml. This serum concentration provides protection in infants from severe disease and protection in cotton rats from RSV infection. In the neutralization assay described above, 40 μg /ml of Palivizumab yields an EC_50_ of 100.

## Results

### DS-Cav1 without foldon is a monomer

**Fig 1:**
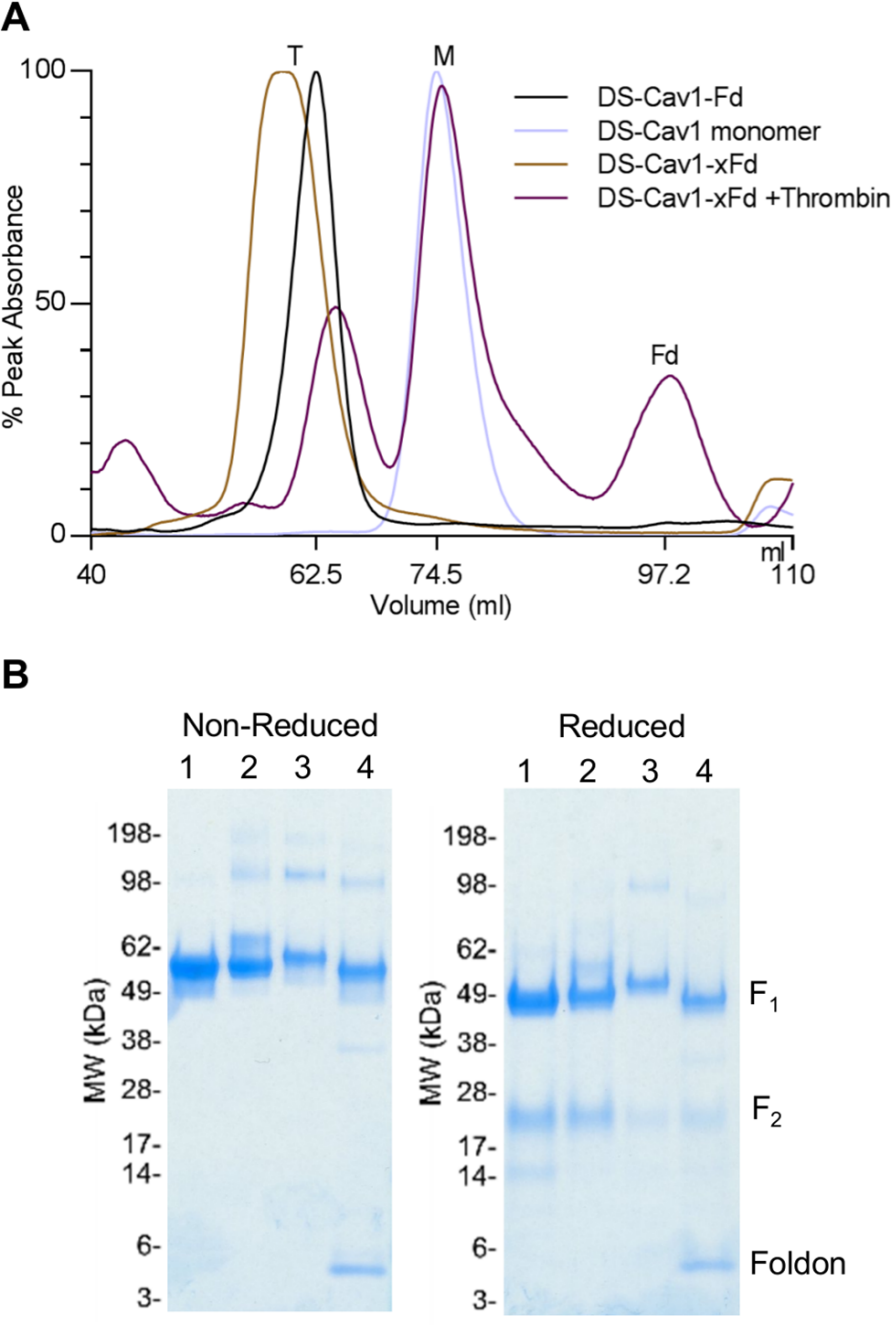
Characteristics of RSV F DS-Cav1 with and without C-terminal phage foldon **(A)** S200 size-exclusion chromatography profile for RSV F DS-Cav1 with and without foldon in the expression construct illustrating the relative positions of purified trimer (T) and monomer (M) and DS-Cav1xFd (DS-Cav1 with a thrombin cleavage site between the α10 helix and the foldon) before and after treatment with thrombin, showing the conversion of trimer to monomer when the foldon is removed. **(B)** SDS-PAGE analysis of (1) DS-Cav1 monomer expressed without foldon, (2) DS-Cav1-foldon (Fd) with C-terminal purification his6 and Strep-tag II tags removed, (3) DS-Cav1-with thrombin cleavable foldon (xFd) and C-terminal purification his6 and Strep-tag II tags (4) DS-Cav1-thrombin cleavable foldon (xFd) after treatment with thrombin.

Each protomer of the trimeric DS-Cav1 spirals around a central 3-fold axis, with a RSV-only (no foldon) interface of over 2000 Å^2^. As this large interface might form a stable trimer, we first tested a DS-Cav1 without foldon, created by introducing a stop codon prior to the C-terminal foldon motif. Expression of this DS-Cav1 without foldon resulted in a substantially lower ratio of D25 to motavizumab ELISA compared to DS-Cav1 with C-terminal foldon (S1 Table); when purified by tandem affinity chromatography and size-exclusion chromatography, DS-Cav1 without foldon eluted at a position corresponding to 55 kDa, substantially after that of DS-Cav1 with foldon, which elutes at a position corresponding to 165 kDa. We also created a DS-Cav1 construct with a thrombin cleavage site inserted between RSV F and foldon motif (DS-Cav1xFd). This construct initially eluted as a trimer of 165 kDa; however, after enzymatic cleavage to remove the foldon, it dissociated into 55 kDa monomers (Fig 1A and 1B).

### DS-Cav1 variants recognized by D25 may be monomeric

We initially attempted to use structure-based design with antigenic selection, to identify a stable trimeric DS-Cav1 variant without foldon. DS-Cav1 variants were designed to retain trimeric F glycoprotein assembly in the absence of a foldon (S1 Table). Because the D25 epitope is partially quaternary [10] and because our initial monomeric DS-Cav1 had poor D25 recognition, we sought to use D25 recognition as a surrogate for trimer formation. Several of the initial designs showed high D25 ELISA binding titers (>2.0 OD) when assessed on 293T transient expression culture supernatants in 96-well microtiter plate format. We expressed two of these designs (DS-Cav1, 505-FIRKSDEWY and DS-Cav1, 505- FIRKSDECEEC; S1 Table) in 1 L cultures; however, on size-exclusion chromatography, both of these variants eluted as 55 kDa monomers. These observations are in agreement with recently published data [39] in which monomeric RSV F has been reported to bind pre-fusion neutralizing antibodies.

### Interprotomer disulfides create stable DS-Cav1 oligomers even after foldon removal

**Fig 2:**
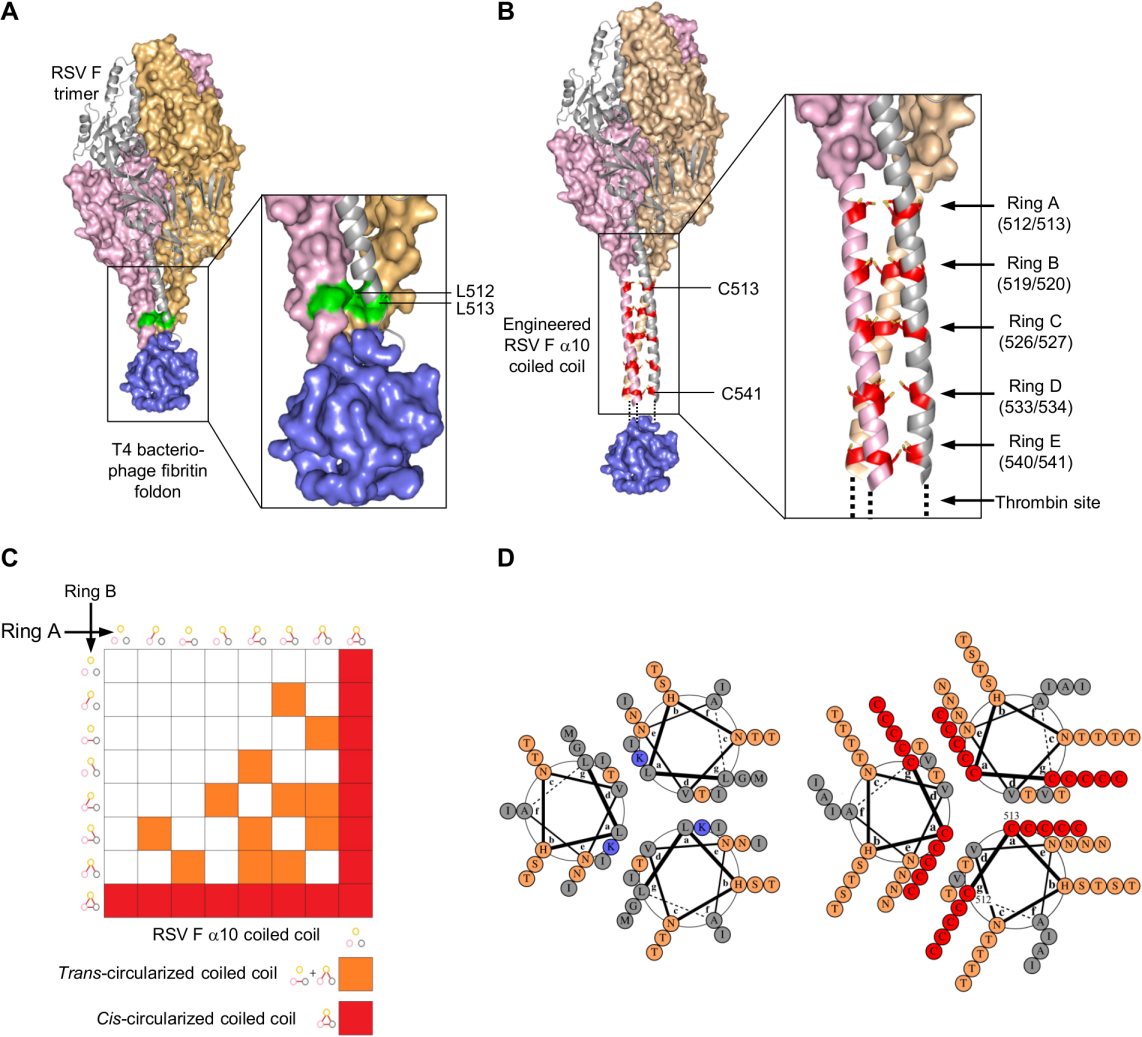
Design of pre-fusion RSV F with inter-protomer disulfide rings within the viral α10 coiled-coil. **(A)** Pre-fusion DS-Cav1 RSV F trimer structure with T4 fibritin foldon trimerization domain colored in blue showing residues L512 and L513 in green. **(B)** Designed pre-fusion RSV F with inter-protomer disulfide rings A-E within the α10 coiled-coil. Pre-fusion DS-Cav1 RSV F trimer structure with extended α10 helix modelled and depiction of cysteines introduced into the coiled-coil core to covalently link the three protomers. **(C)** 2-dimensional complementation grid exemplifying 2 rings where combinations of covalent closure of the coiled-coil by a single pair of cysteines are shown in red (*cis*-circularization) and multiple pairs of cysteines shown in orange (*trans*-circularization) and incomplete covalent circularization of the protomers shown in white. **(D)** Wheel diagram representations of the α10 coiled-coil wild-type (left) and 5-ring engineered coiled-coil (right) as generated by DrawCoil 1.0: http://www.grigoryanlab.org/drawcoil/ [32].

Since we could not use D25 selection to identify stable trimers, we chose to focus on interprotomer disulfides, the formation of which could be assessed by non-reducing SDS-PAGE. The trimeric structure of pre-fusion RSV F [10] contains a membrane-proximal helix (α10), which forms a left-handed coiled-coil with C3 symmetry about the trimer axis with a tilt angle of about 30 degrees (Fig 2). We attempted to stabilize the RSV F trimer by introducing interprotomer disulfide bonds in the RSV F C-terminal coiled-coil that would allow for stable trimers. Because the foldon is attached to the C-terminus of this helix, these interprotomer disulfides would stabilize the trimer at a position similar to that of the foldon in the parent RSV F DS-Cav1+foldon.

Numerical fit of canonical Crick coiled-coil parameters [40] revealed the RSV F helical geometry to have substantial differences from the geometry of a canonical coiled-coil (2.4 Å Cα RMSD fit over residues 592–513). However, inspection of the C-terminal portion of the α10-helices revealed a “pinching in” of the helices, with formation of a more canonical coiled-coil C-terminal of residue 507. In particular, the coiled-coil helices surrounded an aqueous cavity between β23 strand (residue 492) and the inter-helix contact at residue 505, with the helices approaching a suitable distance for side-chain disulfide formation at inward facing “A” and “G” residues of the heptad repeat (Fig 2B). We hypothesized that suitably positioned cysteine pairs in the RSV F α10 coiled-coil would covalently *cis*-circularize protomers about the trimer axis with formation of three disulfide bonds between the three helices in a ring-like structure.

Specifically pairs of mutations L512C/L513C, G519C/K520C and M526C/I527C were separately introduced into the foldon-cleavable DS-Cav1. We refer to mutants with 512C-513C as containing “Ring A”, mutants with 519C-520C as containing “Ring B”, and mutants with 526C-527C as containing “Ring C”.

**Fig 3:**
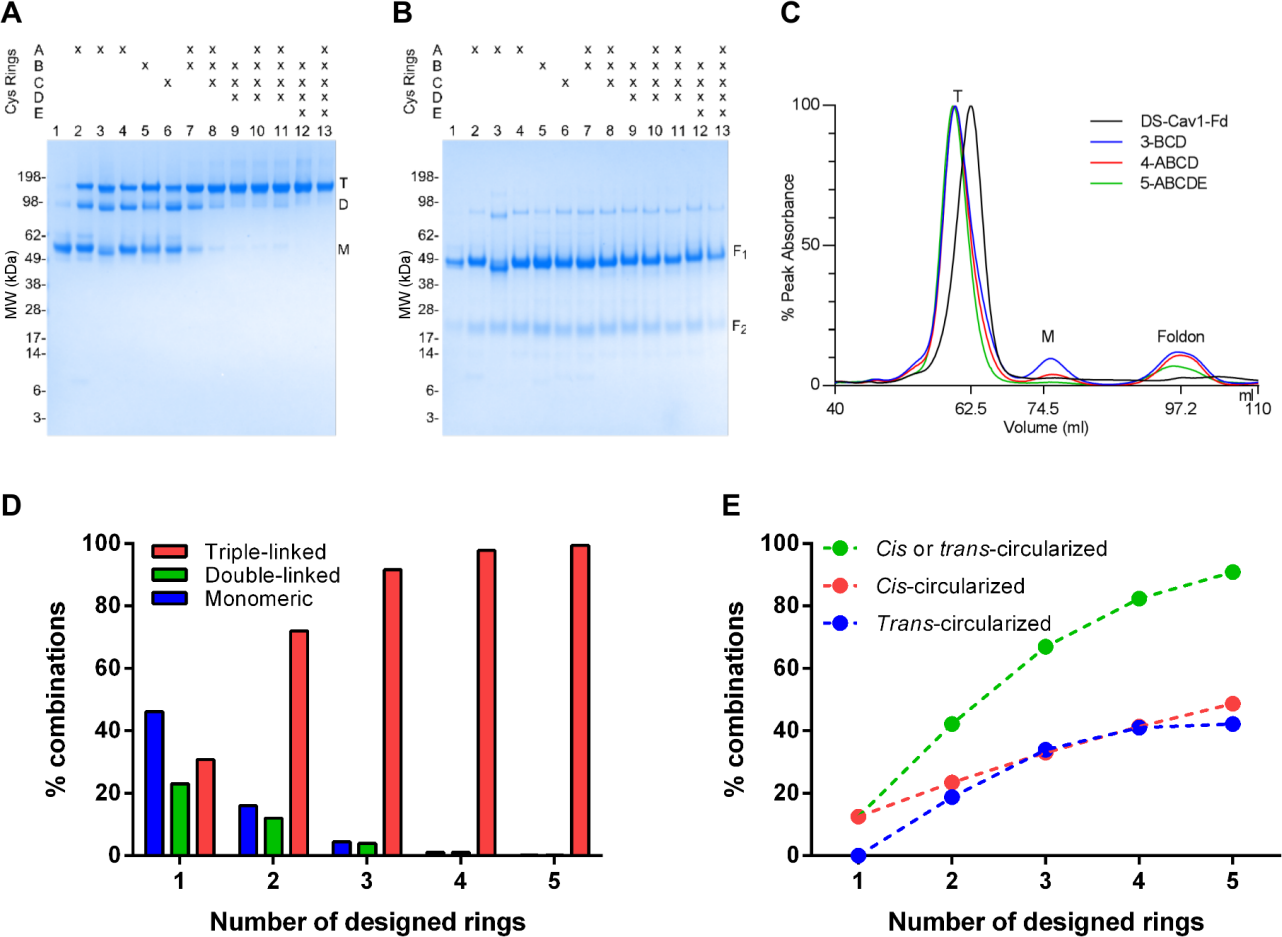
α10 helix cysteines form inter-protomer disulfides that efficiently cross-link RSV F trimers. **(A)** Non-reducing and **(B)** reducing SDS-PAGE of engineered RSV F glycoproteins with a C-terminal, thrombin-cleavable foldon after tandem purification by his_6_ and Strep-tag II tags and visualized by Coomassie stain. Bands corresponding to covalently linked RSV F trimer (T), dimer (D), and RSV F monomer (M) are indicated on panel A. Bands corresponding to RSV polypeptides F_1_ and F_2_ are indicated on panel B. Lanes 1, DS-Cav1-Fd; 2, 1-A; 3, 1-A-F505W+SM; 4, 1-A-long; 5, 1-B; 6, 1-C; 7, 2-AB; 8, 3-ABC; 9, 3-BCD; 10, 4-ABCD-1; 11, 4-ABCD-2; 12, 4-BCDE; 13, 5-ABCDE. **(C)** Size exclusion chromatography of representative engineered RSV F glycoproteins after cleavage of the foldon compared to DS-Cav1-Fd. Peaks corresponding to covalently linked RSV F trimer (T), monomeric thrombin-cleaved RSV F (M), thrombin-cleaved foldon (Fd) are indicated on the chromatogram. **(D)** Calculated relative proportions of triple-linked, double-linked and unlinked monomeric RSV F protomers with one to five disulfide rings in the α10 helices. **(E)** Calculated relative proportions of *cis-* and *trans*-circularized crosslinking based on the assumption of an equal probability of neighboring cysteines in the α10 helices forming a covalent bond between helices of the coiled-coil to create a covalently ‘circularized’ ring within the coiled-coil.

One design (DS-Cav1 1-A-F505W+SM) contained Ring A and additional mutations that were intended to enhance the stability of the α10 coiled-coil by a cavity-filling substitution in the coiled-coil core (F505W) and by mutations at solvent-exposed regions that might stabilize the pre-fusion conformation. Purification of the DS-Cav1 1-A-F505W+SM protein revealed this design to form inter-protomer disulfide bonds as shown by reducing and non-reducing SDS-PAGE (Fig 3A and 3B) and by elution on size-exclusion chromatography at a position corresponding to the predicted trimer molecular weight (165 kDa) (Fig 3C) (Table 1). However, digestion with thrombin and size-exclusion purification revealed a significant fraction (>90%) to separate into RSV F dimers and monomers. Through successive rounds of size-exclusion chromatography, a pure trimeric fraction was isolated and used for antigenic and physical analyses and immunization studies.

**Table 1:** Antigenic and physical characteristics of interprotomer-disulfide stabilized RSV F glycoprotein trimers.^1^

Construct name	α10 coiled coil sequence^2^	Number of cysteine- rings	Purified yield^3^ (mg/L)	Trimeric yield^4^	Site Ø antibody			Physical characteristics^5^						
					K_D_ (nM)			Temp (°C)		pH		Osmolarity (mM)		Freeze-thaw
					D25	AM22	5C4	50	70	3.5	10	10	3000	
**DS-Cav1-Fd [Ref. 18]**	LLSAIGG-Fd-*GGLVPR*	0	1.9	N/A	0.15	<0.01	13	0.9	0.0	0.8	0.9	1.0	0.8	0.7
**0**	LL*GGLVPR*	0	1.6	0%	n.d.	n.d.	n.d.	1.0	1.0	0.6	1.0	0.9	1.0	0.6
**1-A**	**CC**HNVNAGKSTTN*GGLVPR*	1	3.4	8%	n.d.	0.67	26	1.0	0.3	0.9	1.0	1.0	0.8	0.2
**1-B**	LLHNVNA**CC**STTN*GGLVPR*	1	2.7	3%	n.d.	1.08	26	1.0	0.3	0.9	0.9	1.0	0.7	0.3
**1-C**	LLHNVNAGKSTTN**CC**ITT*GGLVPR*	1	2.9	3%	0.32	1.29	30	0.9	0.4	0.9	1.0	1.0	0.7	0.4
**2-AB**	**CC**HNVNA**CC**STTN*GGLVPR*	2	5.5	14%	0.21	0.71	31	0.7	0.6	0.6	0.9	0.8	0.9	0.6
**3-BCD**	LLHNVNA**CC**STTNI**CC**TTTNI**CC**TT*GGLVPR*	3	4	30%	0.38	1.26	30	0.9	1.0	0.9	1.0	0.9	0.9	0.3
**4-ABCD**	**CC**HNVNA**CC**STTNI**CC**TTTNI**CC**TT*GGLVPR*	4	3.7	38%	0.32	1.74	38	1.0	1.0	1.0	1.0	1.0	1.0	0.5
**4-BCDE**	LLHNVNA**CC**STTNI**CC**TTVNA**CC**STTNI**CC**TT*GGLVPR*	4	3.4	15%	0.33	1.9	26	0.9	0.8	0.6	0.8	n.d.	n.d.	0.6
**5-ABCDE**	**CC**HNVNA**CC**STTNI**CC**TTVNA**CC**STTNI**CC**TT*GGLVPR*	5	2.6	24%	0.47	1.1	33	1.0	1.0	1.0	1.0	1.0	1.0	0.4

^1^ Biophysical measurements performed without foldon apart from the DS-Cav1-Fd control.

^2^ Sequence shown stating at residue 512; Fd refers to T4 fibritin foldon; thrombin cleavage site residues shown in italics; cysteine pairs shown in bold.

^3^ Yield after Tandem His and Strep-tag II purification prior to removing the foldon.

^4^ Proportion of purified yield following removal of foldon and gel filtration.

^5^ Fractional D25 reactivity remaining after 1 hour incubation at specified condition or **t**en cycles of freeze-thaw.

N/A is not applicable.

n.d. is not determined.

Another design (“Rings AB”) combined cysteine pairs L512C/L513C and G519C/K520C and showed on non-reducing SDS-PAGE an increased proportion of disulfide-bonded trimers (Fig 3A), suggesting complementation between disulfide ring assemblies could be achieved by increasing the numbers of cysteine pairs at G and A positions in the coiled-coil (Fig 2B–2D). These results indicated that covalent closure around the trimer axis could be compensated through *trans*-circularization of the coiled-coil by addition of additional cysteine pairs at appropriate “G” and “A” positions along the helices (Fig 2C). Overall, we observed good agreement between the predicted number of disulfide bonds (Fig 3D) and those formed between the α10 helices (Fig 3A).

### Immunization with 2-ring disulfide-stabilized DS-Cav1 elicited promising neutralization titers

**Fig 4:**
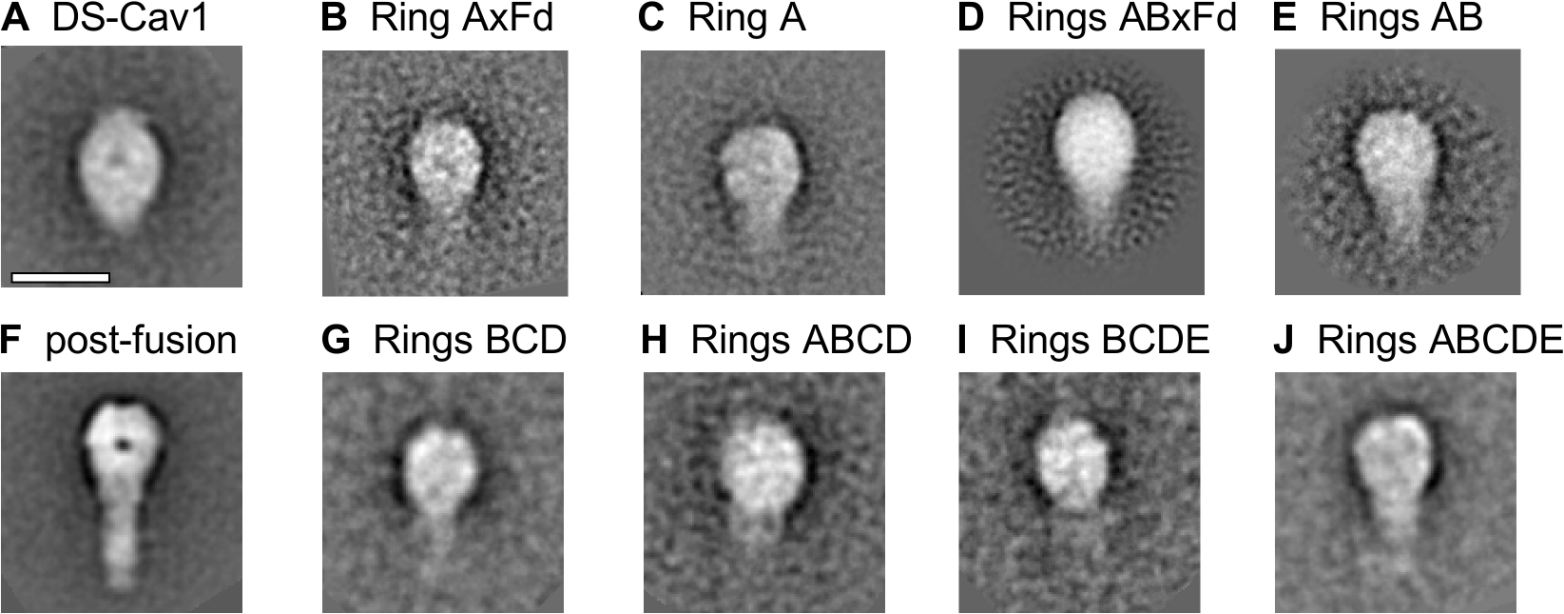
Negative stain-electron microscopy of RSV F glycoproteins stabilized by different C-terminal motifs. **(A-J)** shows typical 2D averaged classes of particles of negatively stained specimens for DS-Cav1 with various disulfide coiled-coil motifs. **(A)** DS-Cav control; **(B)** ring A with foldon; **(C)** ring A without foldon; **(D)** rings AB with foldon; **(E)** rings AB without foldon; **(F)** post-fusion F; **(G)** rings BCD without foldon; **(H)** rings ABCD without foldon; **(I)** rings BCDE without foldon; **(J)** rings ABCDE without foldon. The scale bar is 10 nm.

**Fig 5:**
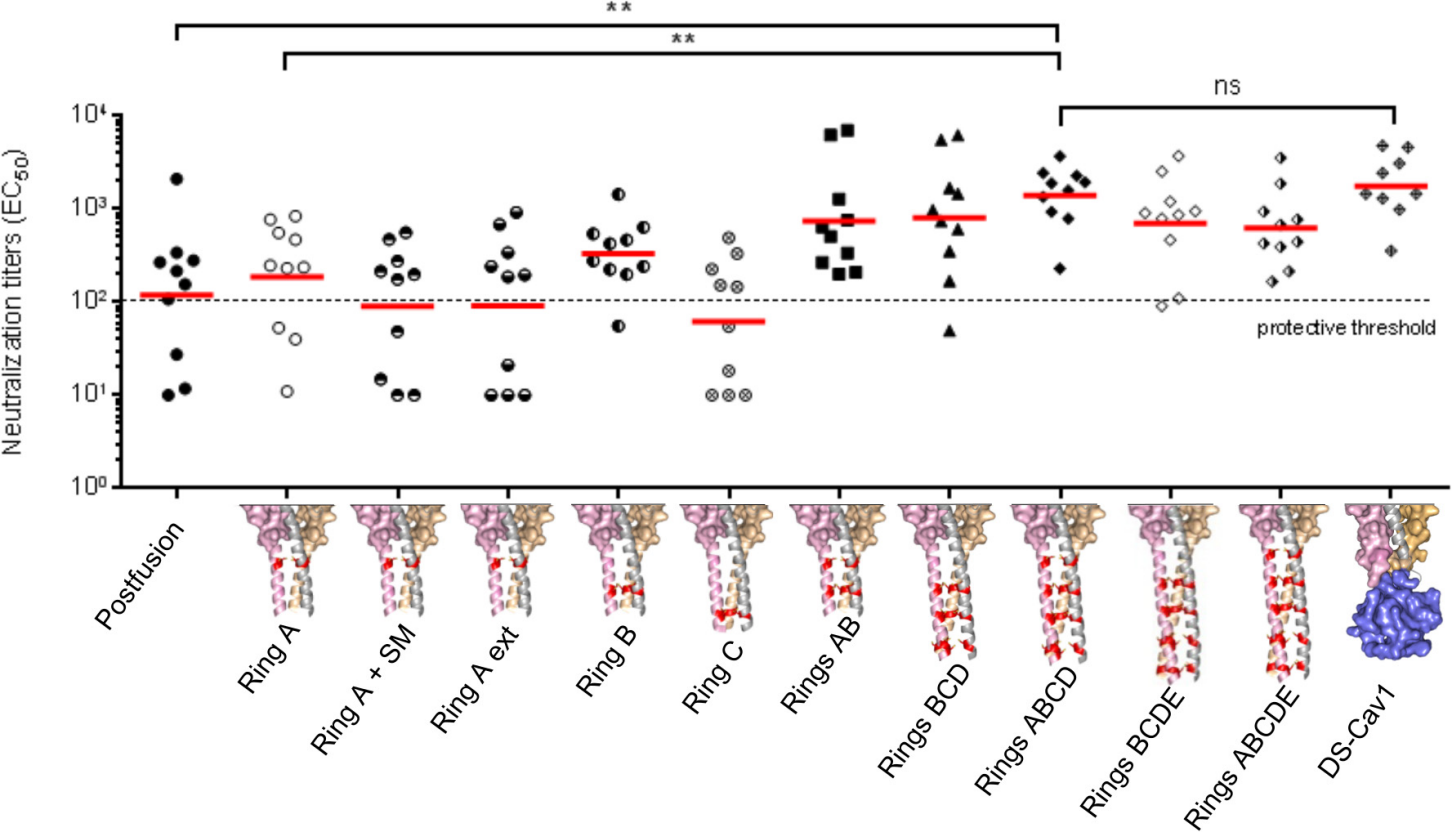
Immunogenicity of inter-protomer disulfide stabilized RSV F variants. Neutralization titers of sera from mice immunized with 10 μg of RSV F variants in the presence of 50 μg of poly I:C adjuvant are shown (10 mice/group). Titers from individual mice are shown as individual colored dots, and the geometric mean of each group is indicated by a red horizontal line. Protective threshold [18] at a neutralization titer of 100 is indicated by a dotted line, and statistical significance as assessed by a Mann-Whitney Unpaired non-parametric two-tailed test followed by false discovery rate correction are shown for post-fusion, ring A, ring ABCD, and DS-Cav1 RSV F immunized groups.

Analysis of the antigenic and physical characteristics of the disulfide linked DS-Cav1 variants after foldon removal and purification of the trimeric species showed broad similarity to the parental DS-Cav1 molecule (Table 1). 1-ring disulfide-linked and 2-ring disulfide linked RSV F trimers were analyzed by negative-stain electron microscopy, to quantify the presence of pre-fusion RSV F trimers (Figs 4A–4J and S1A–S1J). 10μg of each protein were adjuvanted with 50μg poly IC and used to immunize groups of 10 CB6F1/J mice twice with a 3 week interval. Week 5 sera for the 1-ring disulfide linked trimers showed no significant improvement in RSV subtype A neutralization titers compared to post-fusion with reciprocal geometric mean titer (GMT) EC_50_s ranging from 3.5 to 332; however two mice in the “Rings AB” DS-Cav1 stabilized group showed neutralization titers similar to DS-Cav1 (Fig 5) and the 2-ring variant group showed a higher geometric mean titer (GMT) (EC_50_ = 745) than all other 1-ring variants and all mice in this group had sera above the protective threshold (7) (EC_50_ = 100) at week 5.

### Multi-ring cysteine zipper stabilized DS-Cav1 trimers

Although only three sets of rings could be accommodated within the native RSV F coiled-coil, additional rings could be added by duplicating portions of the coiled-coil to create G-A positions at 533C-534C (Ring D) and at 540C-541C (Ring E). Hypothetical calculations for equal levels of disulfide bond formation at 1, 2, 3, 4, and 5 G-A positions in the coiled-coil indicated an asymptotic relationship between the formation of 3-way inter-protomer from *cis-* and *trans*-disulfide circularization and the number of disulfide ring motifs (Fig 3D and 3E). Thus, in light of the promising Rings AB immunogenicity, designs comprising 3, 4, and 5 ring systems were constructed containing combinations of G-A α10 coiled-coil helix mutations in the foldon-cleavable DS-Cav1 (Fig 2B, Table 1). These RSV F variants expressed in Expi293F cells at 2.6–5.5 mg/L, which was higher than reported for DS-Cav1 (Table 1) (7). Reduced and non-reduced SDS-PAGE analysis of these protein variants revealed that, with increasing numbers of cysteine rings in the α-10 helix, an increase in the proportion of covalently associated trimers was observed (Fig 3A and 3B). These multi-ring RSV F glycoproteins, after cleavage of the foldon, retained a main trimer peak following size-exclusion chromatography (SEC) with an absence of any aggregates or higher-order oligomers (Fig 3C). Negative-stain electron microscopy of the foldon-removed, SEC-purified RSV F molecules also confirmed a pre-fusion RSV F trimeric conformation for the particles (Figs 4A–4J and S1A–S1J). Antigenic and physical analyses of these multi-ring RSV F glycoproteins showed that these retained similar characteristics as DS-Cav1; however they were more heat resistant than either DS-Cav1 or the single-cysteine ring containing variants (Table 1).

### High RSV neutralizing activity elicited by cysteine zipper-stabilized DS-Cav1

Immunization of groups of 10 CB6F1/J mice, with the multi-ring DS-Cav1 variants, showed significantly higher RSV neutralization titers than with 1-ring groups, with almost all multi-ring immunized mice displaying levels of RSV neutralizing activity above the protective threshold after 2 immunizations. The 3-ring variant (Rings ABC) had a similar GMT (EC_50_ = 804) to the 2-ring variant (Rings AB); however, the 4-ring variant (Rings ABCD) had the highest geometric mean of all variants (EC_50_ = 1395) and was statistically indistinguishable from DS-Cav1+foldon (EC_50_ = 1762) (Fig 5, S2 Table). Compared to the 4-ring variant ABCD, the GMT EC_50_s of the 4-ring variant BCDE and the 5-ring variant ABCDE were lower (EC_50_s 708 and 623 respectively); however, these groups were not statistically different from DS-Cav1+foldon. Mann-Whitney statistical analysis of all groups compared with DS-Cav1+foldon and post-fusion immunized groups showed the 4 ring variant (Rings ABCD) to be statistically indistinguishable from the DS-Cav1 immunized group (*p* = 0.656) and significantly different from the post-fusion immunized group (*p* = 0.0045) (S3 Table).

## Discussion

Structure-guided vaccine design can enable the identification of modifications of protein antigens to address specific limitations and to advance vaccine candidates to clinical trials. With the RSV F DS-Cav1, removal of the foldon was facilitated by the addition of cysteine-ring motifs to a native C-terminal coiled-coil. The propensity for the RSV F trimer to dissociate into monomers without a trimerization motif despite an extensive inter-protomer surface area could be caused by the inherent large conformational change it undergoes for membrane fusion. It has been shown that disulfides at the membrane-proximal external region (MPER) of paramyxovirus PIV5 F prevent triggering into post-fusion conformation and membrane fusion; this blockage can be reversed in the presence of reducing agent, suggesting the forces between holding the MPER helices together are weak [41]. Attempts to stabilize F in the pre-fusion conformation with inter-protomer disulfide bonds have been previously reported but with different sequences than reported here [42]. The multiple repeating cysteine units in the multi-ring engineered coiled-coil are similar to the leucines in a leucine zipper [43]; however the helices are associated by covalent bonds rather than hydrophobic forces. This ‘cysteine zipper’ allowed for the formation of a highly stable interaction between the three helices over a short number of helical turns. Our work highlights the use of cysteine-stabilized coiled-coils in RSV F trimer stabilization, but there may be other engineering opportunities that could utilize this modality. Coiled-coils have been previously engineered to enhance non-covalent association [44]. A naturally occurring example of a coiled-coil with a *cis*-circularized covalent ring from three pairs of cysteines is the chicken cartilage matrix protein (PDB ID 1AQ5) [45]; in this matrix protein, the disulfide motif (9-CEC) is located at the N-terminus of the coiled-coil, and the motif disrupts the alpha-helical coiled structure of each helix and cannot be transplanted directly into the C-terminal RSV F α10 coiled-coil. An inverse correlation of the quantity of monomeric and dimeric RSV F glycoproteins resolved by size-exclusion chromatography and the number of cysteine rings in the α10 helices was observed, indicating increasing *cis-* and *trans-* complementation of cysteine rings with higher number of rings in the α10 helix. Importantly, once the heterologous T4 foldon domain was removed, several multiple-cysteine ring variants, retained the potent immunogenic characteristics of the parent DS-Cav1, and the ABCD 4-ring variant was capable of eliciting neutralizing titers over ten-fold above the protective threshold level. Although some cysteines are likely unpaired due to the incomplete circularization of the covalent rings, we did not observe evidence of oligomerization of the uncleaved or cleaved multi-cysteine RSV F trimers. Thus unpaired cysteines are either shielded into the core of the coiled-coil or in a non-reactive state.

Sequence modules, comprising purification tags or oligomerization domains, are often added to antigens to enhance processing or immunogenicity during the proof-of-principle stage of vaccine development. However, later stages of vaccine development seek to minimize non-pathogen sequences, to avoid potential safety issues associated with off-target reactivity. Here we show how the commonly used oligomerization domain, the trimeric “foldon” from T4-bacteriophage fibritin, can be removed from this promising recombinant subunit vaccine against RSV.

In summary, we have designed a motif–the cysteine zipper–that robustly stabilizes a coiled-coil such that a heterologous trimerization motif can be removed, allowing for the production of a RSV F DS-Cav1 stabilized antigen that is entirely encoded by the RSV pathogen except for a limited number of stabilizing mutations and the protease recognition residues left after cleavage of the foldon. By systematic assessment of possible engineered coiled-coils for RSV F, and by characterization of immunogenicity *in vivo*, we successfully implemented structure-based rational design to improve a highly promising RSV vaccine candidate for potential use in humans.

## Supporting Information

S1 Checklist Animal Research: Reporting In Vivo Experiments (ARRIVE) guidelines checklist. Page referencing of key issues relating to the animal work performed in this study.
(DOC)

S1 Fig Negative stain-electron microscopy of RSV F glycoproteins stabilized by different C-terminal motifs. **(A-J)** show representative fields of negatively stained specimens for DS-Cav1 with various disulfide coiled-coil motifs. In all specimens a certain amount of particles unfolded on the carbon film and appeared as a more random form which could be monomers linked at their C-termini by the disulfide bonds. The scale bar is 10 nm.
(TIF)

S1 Table Antigenic characteristics of engineered RSV F glycoprotein variants. ELISA binding of RSV F variants transiently expressed and assessed at harvest and following incubation at 4° C for 1 week by site Ø-specific antibody D25 or motavizumab IgG.
(DOCX)

S2 Table Reciprocal serum dilution associated with 50% RSV A2 virus neutralization (EC50) for individual mice at week 5 (two weeks post boost). Designed immunogens without heterologous foldon were assessed for ability to elicit anti-RSV neutralizing antibodies in mice and week 5 neutralization titers are shown.
(DOCX)

S3 Table Statistical analysis of neutralization titers of all immunization groups compared to the postfusion and DS-Cav1 immunized groups. Mann-Whitney Unpaired non-parametric two-tailed test followed by false discovery rate correction. Values < 0.05 (significant at a 5% level) are indicated in italics and values <0.005 (significant at a 0.5% level) are indicated in bold.
(DOCX)
