# Supplementary figures and images for: A Cysteine Zipper Stabilizes a Pre-Fusion F Glycoprotein Vaccine for Respiratory Syncytial Virus
Source: PLoS One. 2015 Jun 22;10(6):e0128779. doi: 10.1371/journal.pone.0128779 (PMC4476739; doi:10.1371/journal.pone.0128779)

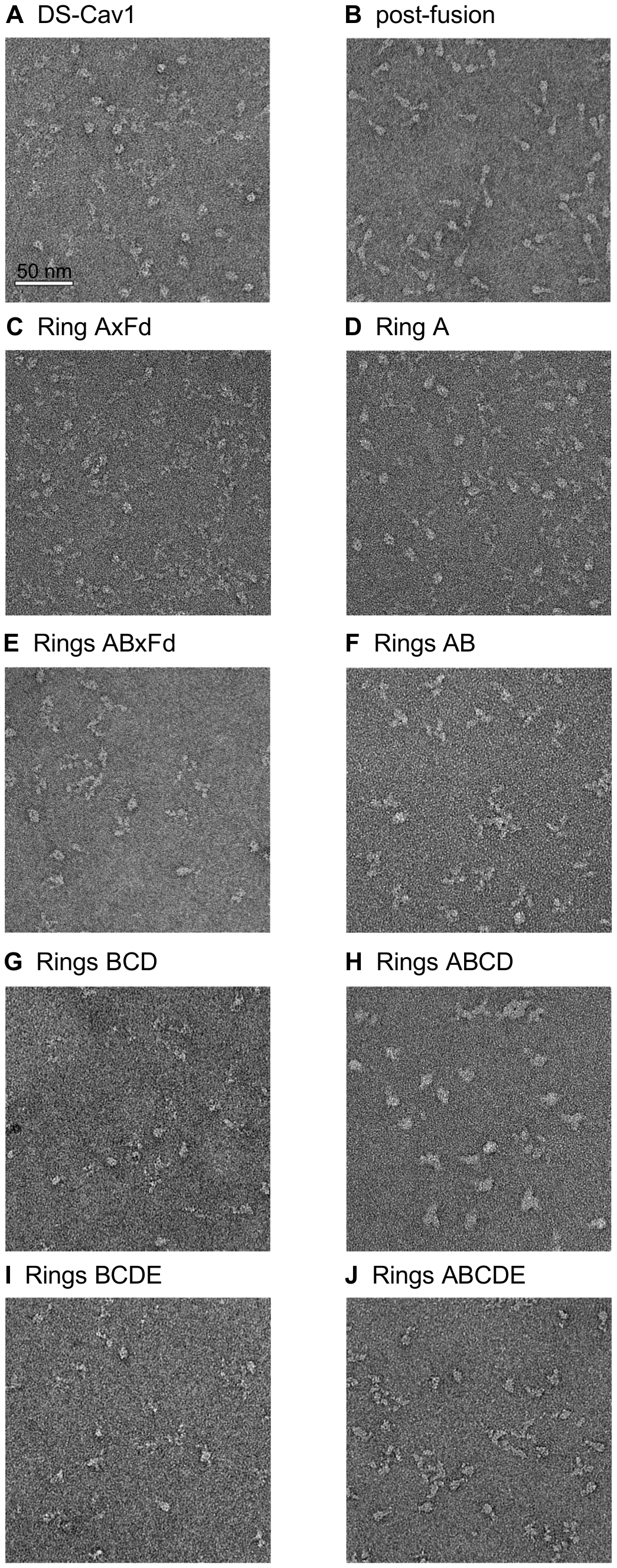

Supplement: S1 Fig — (A-J) show representative fields of negatively stained specimens for DS-Cav1 with various disulfide coiled-coil motifs. In all specimens a certain amount of particles unfolded on the carbon film and appeared as a more random form which could be monomers linked at their C-termini by the disulfide bonds. The scale bar is 10 nm. (TIF) [file pone.0128779.s002.tif]
