# Supplementary material for: A Cysteine Zipper Stabilizes a Pre-Fusion F Glycoprotein Vaccine for Respiratory Syncytial Virus
Source: PLoS One. 2015 Jun 22;10(6):e0128779. doi: 10.1371/journal.pone.0128779 (PMC4476739; doi:10.1371/journal.pone.0128779)
Supplement: S1 Table — ELISA binding of RSV F variants transiently expressed and assessed at harvest and following incubation at 4° C for 1 week by site Ø-specific antibody D25 or motavizumab IgG. (DOCX) [file pone.0128779.s003.docx]

| **S1 Table.** Antigenic characteristics of engineered RSV F glycoprotein variants. | | | | | | | | | |  |
| --- | --- | --- | --- | --- | --- | --- | --- | --- | --- | --- |
| **Mechanism of stabilization** | **RSV F variant^1^** | **ELISA binding^2^ upon expression** | |  | | **ELISA binding^2^ after 1 week at 4 °C** | | | | |
|  |  | **Mota^3^** | **D25** |  | **Mota^3^** | | **D25** | |  |  |
| **None** | RSV F wild type with C-terminal foldon | 2.20 | 2.23 |  | | 2.29 | | 0.29 | | |
|  | DS-Cav1 with C-terminal foldon | 2.63 | 2.57 |  | | 3.08 | | 2.60 | | |
|  | No foldon, no fusion peptide (post-fusion) | 2.11 | 0.30 |  | | 2.97 | | 0.70 | | |
|  | DS-Cav1, No foldon | 1.29 | 0.18 |  | | 2.19 | | 0.26 | | |
| **Cavity filling/repacking/disulfides (inter-protomer) without foldon** | DS-Cav1, 505-FIRKSDEFL | 0.34 | 0.39 |  | | 0.73 | | 0.76 | | |
|  | DS-Cav1, 505-FIRKSDELF | 0.44 | 0.56 |  | | 0.86 | | 0.76 | | |
|  | DS-Cav1, 505-FIRKSDEFF | 0.32 | 0.39 |  | | 0.72 | | 0.62 | | |
|  | DS-Cav1, 505-FIRKSDEYY | 0.43 | 0.49 |  | | 0.94 | | 0.71 | | |
|  | DS-Cav1, 505-FIRKSDEWW | 0.53 | 0.72 |  | | 0.98 | | 0.98 | | |
|  | DS-Cav1, 505-FIRKSDEWY | 1.93 | 2.06 |  | | 2.58 | | 2.33 | | |
|  | DS-Cav1, 505-FIRKWDELL | 0.61 | 0.71 |  | | 0.79 | | 0.80 | | |
|  | DS-Cav1, 505-FIRKFDELL | 0.53 | 0.58 |  | | 0.55 | | 0.53 | | |
|  | DS-Cav1, 505-FIRKWDEFL | 0.72 | 0.74 |  | | 0.74 | | 0.81 | | |
|  | DS-Cav1, 505-FIRKWDEFF | 0.59 | 0.62 |  | | 0.74 | | 0.77 | | |
|  | DS-Cav1, 505-FIRKFDECC | 1.97 | 1.67 |  | | 2.39 | | 1.69 | | |
|  | DS-Cav1, 505-FIRKSDEFCECI | 1.77 | 1.43 |  | | 2.27 | | 1.64 | | |
|  | DS-Cav1, 505-FIRKWDELCECI | 2.33 | 2.23 |  | | 2.43 | | 1.90 | | |
|  | DS-Cav1, 505-FIRKFDELCECI | 1.73 | 1.54 |  | | 2.27 | | 1.74 | | |
|  | DS-Cav1, 505-FIRKWDEFCECI | 1.66 | 1.37 |  | | 2.33 | | 1.66 | | |
|  | DS-Cav1, 505-FIRKSDEFCEEC | 1.53 | 1.21 |  | | 2.28 | | 1.57 | | |
|  | DS-Cav1, 505-FIRKWDELCEEC | 1.51 | 1.18 |  | | 2.16 | | 1.17 | | |
|  | DS-Cav1, 505-FIRKFDELCEEC | 1.25 | 0.93 |  | | 1.98 | | 0.97 | | |
|  | DS-Cav1, 505-FIRKWDEFCEEC | 1.30 | 0.93 |  | | 2.03 | | 0.91 | | |
|  | DS-Cav1, 505-FIRKSDECECG | 1.49 | 1.19 |  | | 2.13 | | 1.34 | | |
|  | DS-Cav1, 505-FIRKSDECEEC | 2.56 | 2.55 |  | | 2.62 | | 2.30 | | |
|  | DS-Cav1, 505-FIRKSDELLSCC | 1.37 | 1.03 |  | | 2.14 | | 1.20 | | |
|  | DS-Cav1, 505-FIRKSDETE | 2.15 | 1.91 |  | | 2.25 | | 1.58 | | |
|  | DS-Cav1, 505-FIRKSDETESCC | 1.43 | 0.89 |  | | 1.92 | | 0.84 | | |
|  | DS-Cav1, 505-FIRKSDESE | 2.05 | 1.64 |  | | 2.30 | | 1.60 | | |
|  | DS-Cav1, 505-FIRKSDESESCC | 1.46 | 1.04 |  | | 1.97 | | 0.82 | | |
|  | DS-Cav1, 505-FIRKSDESD | 2.10 | 1.92 |  | | 2.30 | | 1.56 | | |
|  | DS-Cav1, 505-FIRKSDESDSCC | 1.53 | 1.11 |  | | 1.94 | | 0.84 | | |
|  | DS-Cav1, 505-FIRKSDEFLSCC | 1.98 | 1.53 |  | | 2.42 | | 1.63 | | |
|  | DS-Cav1, 505-FIRKSDELFSCC | 2.14 | 1.70 |  | | 2.30 | | 1.75 | | |
|  | DS-Cav1, 505-FIRKSDEFFSCC | 1.96 | 1.58 |  | | 2.22 | | 1.69 | | |
|  | DS-Cav1, 505-FIRKSDEYYSCC | 1.97 | 1.68 |  | | 2.20 | | 1.53 | | |
|  | DS-Cav1, 505-FIRKSDEFYSCC | 1.97 | 1.81 |  | | 2.37 | | 1.76 | | |
|  | DS-Cav1, 505-FIRKSDEWWSCC | 2.18 | 1.91 |  | | 2.24 | | 1.71 | | |
|  | DS-Cav1, 505-FIRKSDEWYSCC | 1.99 | 1.66 |  | | 2.14 | | 1.55 | | |
|  | DS-Cav1, 505-FIRKWDELLSCC | 1.91 | 1.62 |  | | 2.41 | | 1.75 | | |
|  | DS-Cav1, 505-FIRKFDELLSCC | 2.09 | 1.86 |  | | 2.42 | | 1.76 | | |
|  | DS-Cav1, 505-FIRKWDEFLSCC | 2.07 | 1.83 |  | | 2.43 | | 1.65 | | |
|  | DS-Cav1, 505-FIRKWDEFFSCC | 1.89 | 1.58 |  | | 2.39 | | 1.67 | | |
|  | DS-Cav1, 505-FIRKFDEAASCC | 1.82 | 1.54 |  | | 2.38 | | 1.61 | | |
|  | DS-Cav1, 505-FIRKWDEAASCC | 2.25 | 2.19 |  | | 2.36 | | 1.90 | | |
|  | DS-Cav1, 505-WWRKKFDECC | 2.02 | 1.60 |  | | 2.43 | | 1.53 | | |
|  | DS-Cav1, 505-WWRKKFDELLSCC | 1.69 | 1.39 |  | | 2.21 | | 1.40 | | |
|  | DS-Cav1, 505-WWRKKFDESESCC | 1.97 | 1.85 |  | | 2.48 | | 1.92 | | |

^1^ All mutations were assessed on wild type RSV F with a C-terminal his_6_-StrepTag II tandem purification tag.

^2^ Optical density at 450 nm assessed in a 96-well format as described in Methods.

^3^ Motavizumab (Mota).

| **Table S1 (continued). Antigenic characteristics of engineered RSV F glycoprotein variants.** | | | | | | | |  |
| --- | --- | --- | --- | --- | --- | --- | --- | --- |
| **Mechanism of stabilization** | **RSV F variant^1^** | **ELISA binding^2^ upon expression** | |  | | **ELISA binding^2^ after 1 week at 4 °C** | | |
|  |  | **Mota^3^** | **D25** | |  | **Mota^3^** | **D25** | |
| **Cavity filling/repacking/disulfides (inter-protomer, without foldon)** | DS-Cav1, 505-WWRKKFDEAASCC | 2.03 | 1.76 | |  | 2.25 | 1.39 | |
|  | DS-Cav1, 505-KDRKFDECC | 2.21 | 2.12 | |  | 2.48 | 1.93 | |
|  | DS-Cav1, 505-KDRKFDELLSCC | 2.14 | 1.88 | |  | 2.30 | 1.66 | |
|  | DS-Cav1, 505-KDRKSDECC | 2.16 | 1.88 | |  | 2.39 | 1.65 | |
|  | DS-Cav1, 505-KDRKSDELLSCC | 2.00 | 1.62 | |  | 2.23 | 1.68 | |
|  | DS-Cav1, 505-KDRKSDEAASCC | 2.20 | 2.02 | |  | 2.23 | 1.60 | |
|  | DS-Cav1, 505-KDRKSDESESCC | 2.26 | 1.78 | |  | 2.41 | 1.46 | |
|  | DS-Cav1, 505-KDRKSDESDSCC | 1.90 | 1.63 | |  | 2.30 | 1.38 | |
| **Modified/extended coiled coils, without foldon** | DS-Cav1, 505-FIRKSDEIVHFQNAVESTINTLQTTLEAVAQAI | 1.03 | 0.56 | |  | 1.21 | 0.65 | |
|  | DS-Cav1, 505-FIRKSDELLHNVNAGKSTTN | 1.68 | 1.18 | |  | 1.83 | 1.14 | |
|  | DS-Cav1, 505-FIRKSDELLHNVNAGKSTTNIMITTII | 1.00 | 0.69 | |  | 1.98 | 0.94 | |
|  | DS-Cav1, 505-FIRKSDEIEHKINAILSTQNHIETTIARIK | 2.64 | 2.68 | |  | 2.77 | 2.05 | |
| **Cavityfilling/repacking/disulfides (inter-protomer) and cleavable foldon** | S190F, S155C, S290C, F488W, 505-FIRKSDELCEC + cleavable foldon | 2.40 | 2.37 | |  | 2.80 | 2.57 | |
|  | S190F, S155C, S290C, F488W, 505-FIRKSDELCEEC + cleavable foldon | 2.34 | 2.24 | |  | 2.73 | 2.56 | |
|  | S190F, S155C, S290C, F488W, 505-FIRKSDECEC + cleavable foldon | 2.39 | 2.42 | |  | 2.83 | 2.59 | |
|  | S190F, S155C, S290C, F488W, 505-FIRKSDECEEC + cleavable foldon | 2.27 | 2.22 | |  | 2.76 | 2.52 | |
|  | S190F, S155C, S290C, A424C, V450C, L171C, K191C, F488W, 505-FIRKSDELCEC + cleavable foldon | 0.10 | 0.08 | |  | 0.17 | 0.12 | |
|  | S190F, S155C, S290C, A424C, V450C, L171C, K191C, F488W, 505-FIRKSDELCEEC + cleavable foldon | 0.10 | 0.08 | |  | 0.16 | 0.12 | |
|  | S190F, S155C, S290C, A424C, V450C, L171C, K191C, F488W, 505-FIRKSDECEC + cleavable foldon | 0.12 | 0.08 | |  | 0.17 | 0.11 | |
|  | S190F, S155C, S290C, A424C, V450C, L171C, K191C, F488W, 505-FIRKSDECEEC + cleavable foldon | 0.09 | 0.07 | |  | 0.16 | 0.11 | |
|  | K77C, I217C, S190F, S155C, S290C, A424C, V450C, L171C, K191C, F488W, 505-FIRKSDELCEC + cleavable foldon | 0.09 | 0.07 | |  | 0.15 | 0.08 | |
|  | K77C, I217C, S190F, S155C, S290C, A424C, V450C, L171C, K191C, F488W, 505-FIRKSDELCEEC + cleavable foldon | 0.10 | 0.09 | |  | 0.18 | 0.09 | |
|  | K77C, I217C, S190F, S155C, S290C, A424C, V450C, L171C, K191C, F488W, 505-FIRKSDECEC + cleavable foldon | 0.11 | 0.09 | |  | 0.14 | 0.08 | |
|  | K77C, I217C, S190F, S155C, S290C, A424C, V450C, L171C, K191C, F488W, 505-FIRKSDEC + cleavable foldon | 0.11 | 0.10 | |  | 0.12 | 0.08 | |
|  | S155C, S290C, 505-FIRKSDELCEC + cleavable foldon | 2.35 | 1.97 | |  | 2.75 | 1.96 | |
|  | S155C, S290C, 505-FIRKSDELCEEC + cleavable foldon | 2.10 | 1.62 | |  | 2.67 | 2.12 | |
|  | S155C, S290C, 505-FIRKSDECEC + cleavable foldon | 2.29 | 2.02 | |  | 2.80 | 2.17 | |
|  | S155C, S290C, 505-FIRKSDECEEC + cleavable foldon | 2.18 | 1.71 | |  | 2.74 | 2.20 | |
|  | DS-Cav1, 505-FIRKSDEFL + cleavable foldon | 2.10 | 2.40 | |  | 2.80 | 2.52 | |
|  | DS-Cav1, 505-FIRKSDELF + cleavable foldon | 2.32 | 2.40 | |  | 2.92 | 2.59 | |
|  | DS-Cav1, 505-FIRKSDEFF + cleavable foldon | 2.10 | 2.23 | |  | 2.78 | 2.52 | |
|  | DS-Cav1, 505-FIRKSDEWW + cleavable foldon | 1.94 | 1.90 | |  | 2.49 | 2.24 | |
|  | DS-Cav1, 505-FIRKWDELL + cleavable foldon | 1.94 | 1.96 | |  | 2.45 | 2.02 | |
|  | DS-Cav1, 505-FIRKFDELL + cleavable foldon | 1.45 | 1.47 | |  | 1.94 | 1.58 | |
|  | DS-Cav1, 505-FIRKWDEFL + cleavable foldon | 1.39 | 1.47 | |  | 1.86 | 1.64 | |
|  | DS-Cav1, L160K, V178T, L258K, V384T, I431S, L467Q, 505-FIRKSDELL + cleavable foldon | 0.15 | 0.25 | |  | 0.18 | 0.54 | |
|  | DS-Cav1, F477K, L481Q, V482K, L503Q, 505-FKRKSDELL + cleavable foldon | 2.17 | 1.95 | |  | 2.87 | 2.27 | |
|  | DS-Cav1, L160K, V178T, L258K, V384T, I431S, L467Q, F477K, L481Q, V482K, L503Q, DS-Cav1, 505-FKRKSDELL + cleavable foldon | 0.44 | 1.05 | |  | 0.34 | 1.27 | |
|  | DS-Cav1, 505-FIRKSDECC + cleavable foldon | 2.29 | 2.37 | |  | 2.85 | 2.61 | |
|  | DS-Cav1, L160K, V178T, L258K, V384T, I431S, L467Q, 505-FIRKSDECC + cleavable foldon | 2.02 | 2.08 | |  | 1.84 | 1.38 | |
|  | DS-Cav1, F477K, L481Q, V482K, L503Q, 505-FKRKSDECC + cleavable foldon | 2.35 | 2.44 | |  | 2.63 | 2.08 | |
|  | DS-Cav1, L160K, V178T, L258K, V384T, I431S, L467Q, F477K, L481Q, V482K, L503Q, 505-FKRKSDECC + cleavable foldon | 2.40 | 1.20 | |  | 2.84 | 1.20 | |
|  | DS-Cav1, 505-WIRKSDELL + cleavable foldon | 2.15 | 2.28 | |  | 2.72 | 2.50 | |
|  | DS-Cav1, F505W, L160K, V178T, L258K, V384T, I431S, L467Q, 505-WIRKSDELL + cleavable foldon | 0.22 | 0.56 | |  | 0.19 | 0.60 | |
|  | DS-Cav1, F505W, F477K, L481Q, V482K, L503Q, 505-WKRKSDELL + cleavable foldon | 2.22 | 1.98 | |  | 2.75 | 2.25 | |

^1^ All mutations were assessed on wild type RSV F with a C-terminal his_6_-StrepTag II tandem purification tag.

^2^ Optical density at 450 nm assessed in a 96-well format as described in Methods.

^3^ Motavizumab (Mota).

| **Table S1 (continued). Antigenic characteristics of engineered RSV F glycoprotein variants.** | | | | | | |
| --- | --- | --- | --- | --- | --- | --- |
| **Mechanism of stabilization** | **RSV F variant^1^** | **ELISA binding^2^ upon expression** | |  | **ELISA binding^2^ after 1 week at 4 °C** | |
|  |  | **Mota^3^** | **D25** |  | **Mota^3^** | **D25** |
| **Cavity filling/repacking/disulfides (inter-protomer) and cleavable foldon** | DS-Cav1, F505W, L160K, V178T, L258K, V384T, I431S, L467Q, F477K, L481Q, V482K, L503Q, 505-WKRKSDELL + cleavable foldon | 0.31 | 0.92 |  | 0.30 | 1.06 |
|  | DS-Cav1, 505-WIRKSDECC + cleavable foldon | 2.37 | 2.54 |  | 2.85 | 2.51 |
|  | DS-Cav1, L512C, L513C, F505W, L160K, V178T, L258K, V384T, I431S, L467Q, 505-WIRKSDECC + cleavable foldon | 0.28 | 0.59 |  | 0.29 | 0.80 |
|  | DS-Cav1, L512C, L513C, F505W, F477K, L481Q, V482K, L503Q, 505-WKRKSDECC + cleavable foldon | 2.24 | 2.02 |  | 2.87 | 2.28 |
|  | DS-Cav1, L512C, L513C, F505W, L160K, V178T, L258K, V384T, I431S, L467Q, F477K, L481Q, V482K, L503Q, 505-WKRKSDECC + cleavable foldon | 0.36 | 0.88 |  | 0.58 | 1.53 |
|  | DS-Cav1, 505-505-FIRKSDECC + cleavable foldon | 2.36 | 2.38 |  | 2.79 | 2.60 |
|  | DS-Cav1, D486C, E487P, F488C + cleavable foldon | 2.09 | 2.22 |  | 2.49 | 2.39 |
|  | S155C, S290C, S190F, V207L, L503E, I506K, L230F, L158F, 505-FKRKFDELL + cleavable foldon | 1.00 | 0.90 |  | 1.10 | 0.97 |
|  | S155C, S290C, S190F, V207L, L83F, V90F, 505-FKRKFDELL + cleavable foldon | 2.19 | 2.22 |  | 2.61 | 1.97 |
|  | S155C, S290C, S190F, V207L, L83F, V90F, L230F, L158F, 505-FKRKFDELL + cleavable foldon | 0.70 | 0.69 |  | 0.86 | 0.79 |
|  | S155C, S290C, S190F, V207L, L83F, V90F, L230F, V185F, T54A, 505-WKRKFDELL + cleavable foldon | 0.21 | 0.15 |  | 0.21 | 0.11 |
|  | S155C, S290C, S190F, V207L, L83F, V90F, L230F, I395F, 505-FIRKSDELL + cleavable foldon | 0.94 | 0.19 |  | 0.96 | 0.44 |
|  | S155C, S290C, S190F, V207L, L83F, V90F, L230F, L158F, I395F, V185F, T54A, 505-WKRKFDELL + cleavable foldon | 0.09 | 0.09 |  | 0.09 | 0.09 |

^1^ All mutations were assessed on wild type RSV F with a C-terminal his_6_-StrepTag II tandem purification tag.

^2^ Optical density at 450 nm assessed in a 96-well format as described in Methods.

^3^ Motavizumab (Mota).
