# Supplementary material for: A Cysteine Zipper Stabilizes a Pre-Fusion F Glycoprotein Vaccine for Respiratory Syncytial Virus
Source: PLoS One. 2015 Jun 22;10(6):e0128779. doi: 10.1371/journal.pone.0128779 (PMC4476739; doi:10.1371/journal.pone.0128779)
Supplement: S2 Table — Designed immunogens without heterologous foldon were assessed for ability to elicit anti-RSV neutralizing antibodies in mice and week 5 neutralization titers are shown. (DOCX) [file pone.0128779.s004.docx]

**S2 Table.** Reciprocal serum dilution associated with 50% RSV A2 virus neutralization (EC50) for individual mice at week 5 (two weeks post boost).

| Postfusion | Ring A | Ring A + SM | Ring A ext | Ring B | Ring C | Ring AB | Ring BCD | Ring ABCD | Ring BCDE | Ring ABCDE | DS-Cav1 |
| --- | --- | --- | --- | --- | --- | --- | --- | --- | --- | --- | --- |
| 2113.00 | 236.40 | 176.10 | 339.60 | 464.90 | <10 | 1285.00 | 49.52 | 1351.00 | 90.79 | 776.20 | 1465.00 |
| 155.50 | 844.00 | 48.60 | <10 | 242.10 | <10 | 6235.00 | 352.20 | 229.60 | 2533.00 | 166.10 | 4624.00 |
| 268.10 | 53.00 | 214.50 | <10 | 424.70 | 490.50 | 200.20 | 611.60 | 2450.00 | 464.40 | 944.60 | 1308.00 |
| 340.10 | 230.90 | 472.20 | 242.40 | 278.70 | 18.20 | 759.50 | 978.70 | 1895.00 | 3740.00 | 677.30 | 990.70 |
| 10.00 | 247.70 | <10 | <10 | 55.60 | 226.90 | 506.90 | 1464.00 | 1588.00 | 911.10 | 393.20 | 4799.00 |
| 11.80 | 40.00 | 559.80 | 685.70 | 542.30 | 332.10 | 6988.00 | 747.00 | 2282.00 | 110.00 | 3559.00 | 357.00 |
| 107.20 | 469.80 | 198.20 | 187.10 | 1441.00 | <10 | 338.30 | 1691.00 | 1940.00 | 865.00 | 1877.00 | 1458.00 |
| 27.40 | 11.00 | 14.80 | 925.20 | 198.10 | 54.90 | 268.20 | 6233.00 | 938.00 | 793.00 | 428.00 | 2446.00 |
| 216.60 | 773.50 | 10.00 | 195.20 | 225.10 | 146.50 | 209.50 | 5547.00 | 794.00 | 952.00 | 214.00 | 3113.00 |
| 280.30 | 557.30 | 276.40 | 21.10 | 637.70 | 151.70 | 643.70 | 169.00 | 3707.00 | 1204.00 | 446.00 |  |
