# Supplementary material for: A Cysteine Zipper Stabilizes a Pre-Fusion F Glycoprotein Vaccine for Respiratory Syncytial Virus
Source: PLoS One. 2015 Jun 22;10(6):e0128779. doi: 10.1371/journal.pone.0128779 (PMC4476739; doi:10.1371/journal.pone.0128779)
Supplement: S3 Table — Mann-Whitney Unpaired non-parametric two-tailed test followed by false discovery rate correction. Values < 0.05 (significant at a 5% level) are indicated in italics and values <0.005 (significant at a 0.5% level) are indicated in bold. (DOCX) [file pone.0128779.s005.docx]

**S3 Table.** Statistical analysis^1^ of neutralization titers of all immunization groups compared to the postfusion^2^ and DS-Cav1 immunized groups.

|  | Post-fusion | DS-Cav1 |
| --- | --- | --- |
| Postfusion | X | 0.0017 |
| Ring A | 0.4999 | 0.0016 |
| Ring A + SM | 0.7556 | 0.0007 |
| Ring A ext | 0.7041 | 0.0007 |
| Ring B | 0.1462 | 0.0035 |
| Ring C | 0.3879 | 0.0007 |
| Rings AB | *0.0353* | 0.1179 |
| Rings BCD | *0.0343* | 0.3155 |
| Rings ABCD | **0.0045** | 0.6560 |
| Rings BCDE | *0.0353* | 0.0701 |
| Rings ABCDE | *0.0302* | 0.0488 |
| DS-Cav1 | **0.0017** | X |

^1^ Mann-Whitney Unpaired non-parametric two-tailed test followed by false discovery rate correction.

^2^ Values < 0.05 (significant at a 5 % level) are indicated in italics and values <0.005 (significant at a 0.5 % level) are indicated in bold.
